# Need of preventive photocoagulation for retinal arterial macroaneurysm with retinal hemorrhage

**DOI:** 10.1002/ccr3.5683

**Published:** 2022-04-05

**Authors:** Daisuke Nagasato, Takuji Iwawaki, Hitoshi Tabuchi

**Affiliations:** ^1^ 157629 Department of Ophthalmology Tsukazaki Hospital Himeji Japan; ^2^ Department of Technology and Design Thinking for Medicine Hiroshima University Graduate School Hiroshima Japan; ^3^ Iwawaki Eye Clinic Asago Japan

**Keywords:** photocoagulation, retinal arterial macroaneurysm, retinal hemorrhage, vitreous hemorrhage

## Abstract

Retinal arterial macroaneurysm shows rapid vision loss when rupture occurs; therefore, preventive photocoagulation should be considered, if necessary.

## Graphical abstract

Preventive photocoagulation for retinal arterial macroaneurysm with retinal hemorrhage might have prevented vitreous hemorrhage.

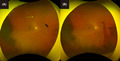

## 1 CASE DESCRIPTION

**FIGURE 1:**
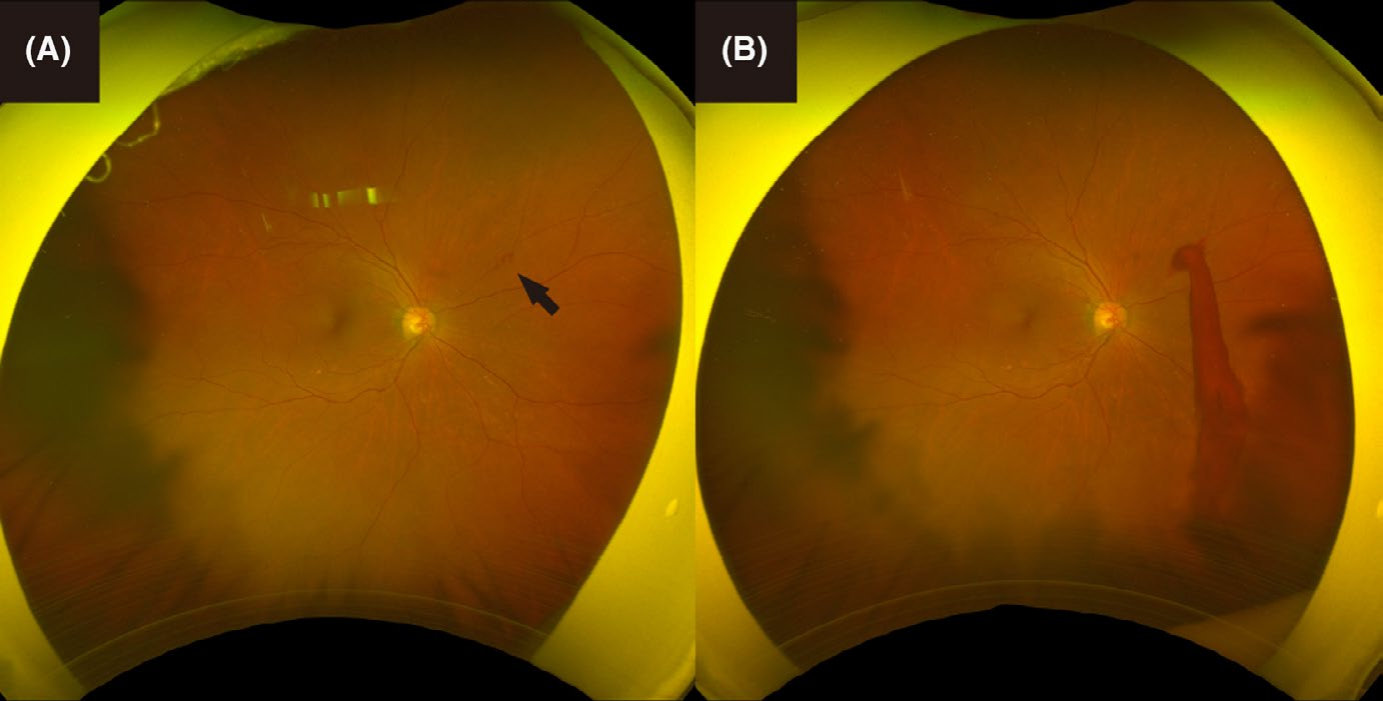
Ultra‐wide‐field pseudo‐color fundus images of the patient's right eye. Retinal arterial microaneurysm (RAM) and mild retinal hemorrhage were revealed in superior‐nasal retinal artery at an annual ophthalmologic examination (A, arrow). After five days, vitreous hemorrhage occurred from the RAM rupture (B)

A 66‐year‐old female patient underwent an annual ophthalmologic examination by her family doctor. At her last visit, fundus examination revealed a retinal arterial macroaneurysm (RAM) and mild retinal hemorrhage of the right eye on ultra‐wide‐field pseudo‐color fundus images (Figure 1A). She had no visual symptoms; therefore, her family doctor ordered a follow‐up visit. Five days later, she returned due to the feeling of a strong floater in her right eye. Vitreous hemorrhage associated with the RAM was observed (Figure 1B). Her best‐corrected visual acuity (BCVA) was 20/40. Vitrectomy was performed for removing the vitreous hemorrhage, and a laser was applied to the RAM. After the vitrectomy, her BCVA improved to 20/20.

There are many treatments for RAM, but no standard treatment protocol has been established. Most RAMs have a benign course of thrombosis, fibrosis, and spontaneous resolution, and the vision returns to its previous state.^1^ Therefore, RAM with no symptoms is generally followed up. When edema or exudates due to RAM cause vision loss, photocoagulation is usually considered.^2^ Photocoagulation may be performed directly on macroaneurysms to facilitate involution.

Preventive photocoagulation for the RAM with retinal hemorrhage in the annual ophthalmologic examination might have prevented vitreous hemorrhage.

## CONFLICT OF INTEREST

There are no conflicts of interest.

## AUTHOR CONTRIBUTIONS

Daisuke Nagasato, MD, PhD has managed the patient; written the manuscript; and critically reviewed the manuscript, references, and images. Takuji Iwawaki, MD has managed the patient. Hitoshi Tabuchi, MD, PhD, EMBA has reviewed the manuscript.

## ETHICAL APPROVAL

The authors have no ethical conflicts to disclose.

## CONSENT

Written informed consent was obtained from the patient to publish this report in accordance with the journal's patient consent policy.

## Data Availability

The data that support the findings of this study are available on request from the corresponding author. The data are not publicly available due to privacy or ethical restrictions.
